# Using Nutrition for Intervention and Prevention against Environmental Chemical Toxicity and Associated Diseases

**DOI:** 10.1289/ehp.9549

**Published:** 2007-01-16

**Authors:** Bernhard Hennig, Adrienne S. Ettinger, Ronald J. Jandacek, Sung Koo, Craig McClain, Harold Seifried, Allen Silverstone, Bruce Watkins, William A. Suk

**Affiliations:** 1 Molecular and Cell Nutrition Laboratory, College of Agriculture, University of Kentucky, Lexington, Kentucky, USA; 2 Department of Environmental Health, Harvard School of Public Health, Boston, Massachusetts, USA; 3 Department of Pathology and Laboratory Medicine, University of Cincinnati, Cincinnati, Ohio, USA; 4 Department of Nutritional Sciences, University of Connecticut, Storrs, Connecticut, USA; 5 Department of Medicine, Division of Gastroenterology/Hepatology, University of Louisville, Louisville, Kentucky, USA; 6 National Cancer Institute, National Institutes of Health, Department of Health and Human Services, Rockville, Maryland, USA; 7 Department of Microbiology and Immunology, SUNY Upstate Medical University, Syracuse, New York, USA; 8 Lipid Chemistry & Molecular Biology Laboratory, Department of Food Science, Purdue University, West Lafayette, Indiana, USA; 9 Center for Risk and Integrated Sciences, National Institute of Environmental Health Sciences, National Institutes of Health, Department of Health and Human Services, Research Triangle Park, North Carolina, USA

**Keywords:** antioxidants, diet, disease, environmental toxicants, nutrition, pollutants, prevention

## Abstract

**Background:**

Nutrition and lifestyle are well-defined modulators of chronic diseases. Poor dietary habits (such as high intake of processed foods rich in fat and low intake of fruits and vegetables), as well as a sedentary lifestyle clearly contribute to today’s compromised quality of life in the United States. It is becoming increasingly clear that nutrition can modulate the toxicity of environmental pollutants.

**Objectives:**

Our goal in this commentary is to discuss the recommendation that nutrition should be considered a necessary variable in the study of human disease associated with exposure to environmental pollutants.

**Discussion:**

Certain diets can contribute to compromised health by being a source of exposure to environmental toxic pollutants. Many of these pollutants are fat soluble, and thus fatty foods often contain higher levels of persistent organics than does vegetable matter. Nutrition can dictate the lipid milieu, oxidative stress, and antioxidant status within cells. The modulation of these parameters by an individual’s nutritional status may have profound affects on biological processes, and in turn influence the effects of environmental pollutants to cause disease or dysfunction. For example, potential adverse health effects associated with exposure to polychlorinated biphenyls may increase as a result of ingestion of certain dietary fats, whereas ingestion of fruits and vegetables, rich in antioxidant and anti-inflammatory nutrients or bioactive compounds, may provide protection.

**Conclusions:**

We recommend that future directions in environmental health research explore this nutritional paradigm that incorporates a consideration of the relationships between nutrition and lifestyle, exposure to environmental toxicants, and disease. Nutritional interventions may provide the most sensible means to develop primary prevention strategies of diseases associated with many environmental toxic insults.

There is clear evidence that exposure to environmental chemicals or pollutants can contribute to compromised health and the pathology of many age-related diseases (Delfino et al. 2005; Hennig et al. 2005; Needham et al. 2005). Most human exposure involving toxic chemicals or mixtures appear to originate from environmental and occupational sources [Centers for Disease Control and Prevention (CDC) 2005; Schafer and Kegley 2002]. Uncontrolled hazardous waste sites and the ever-increasing use and accumulation of chemical pollutants, particularly persistent organic pollutants (POPs), are a major environmental and public health concern in the United States.

In most Western countries, including the United States, diet-related chronic diseases represent the single largest cause of morbidity and mortality. Conditions such as cardiovascular diseases, diabetes, and obesity have reached epidemic proportions in populations of mostly developed nations, but are still rare or nonexistent among people who rely on food through hunting/gathering or individual farming. Research over the last several decades clearly indicates that the pathology of virtually all age-related or chronic diseases (sometimes referred to “diseases of civilization”) is regulated by multifactorial elements that include diet, exposure to environmental agents, and genetic susceptibility (Cordain et al. 2005).

To define the state of the science that supports consideration of a nutritional paradigm in environmental health sciences, the University of Kentucky Superfund Basic Research Program convened a group of experts in the fields of nutritional sciences, medicine, and environmental toxicology for a workshop on “Nutrition and Environmental Chemical Toxicity” on 18 November 2005 at the University of Kentucky. An important outcome of this meeting was the reinforcement that there is a growing need to further understand the complex interplay between environmental exposure, nutrition, and disease risk. The importance of this research was underscored in remarks made by David A. Schwartz in a recent news release [National Institute of Environmental Health Sciences (NIEHS) 2006]:

We need better tools to evaluate environmental exposures, dietary intake and activity levels, and then to determine how those risk factors interact with specific genotypes to either maintain health or lead to disease.

## Discussion

There is no easy “fix” to protect or intervene against diseases associated with exposure to environmental pollutants. Many pollutants, such as heavy metals and persistent organics, bioaccumulate in our bodies, and remediation strategies to remove these chemicals from the environment are extremely difficult and costly. Furthermore, many environmental pollutants induce signaling pathways that respond to oxidative stress; these same pathways are associated with the etiology and early pathology of many chronic diseases (Hennig et al. 2005). Therefore, strategies that modulate the effect of toxicants on pathophysiologic processes involved in disease etiology and progression will be of public health importance.

Evolving studies, derived from epidemiologic and basic research and clinical data, suggest that diet or nutrition, as well as lifestyle changes, can modify pathologies of chronic diseases, as well as diseases associated with environmental toxic insults. For example, the aryl hydrocarbon receptor (AhR) has been the focus of extensive studies in environmental toxicology, and toxic effects of dioxin and dioxin-like compounds are mediated by activation of the AhR (Mandal 2005; Staples et al. 1998). In contrast, many endogenous and exogenous compounds (including dietary components) that have been identified as having agonist or reverse-agonist properties also mediate their effects by activating the AhR, yet these compounds do not exhibit toxicity (Zhou et al. 2003). Furthermore, recent studies suggest that several AhR-activating compounds affect the immune response in a beneficial way by inhibiting symptoms of allergies and asthma (Negishi et al. 2005). A partial explanation for these seemingly dichotomous functions may lie in how the AhR is activated. Studies performed by Nagai et al. (2006) suggest that dioxin-like compounds that cause the AhR to be permanently active lead to toxic effects, whereas dietary components that promote temporal activation, without persistent binding of the AhR/AhR nuclear translocator complex to dioxin response element sequences, might avoid toxic effects while promoting beneficial effects. More research is needed into how various ligands activate the AhR, and how this can result in variable and clinical relevant outcomes.

Another example in which nutritional intervention may play a role in improving health is in the interaction of industrial toxicants with nonalcoholic fatty liver disease (NAFL) or nonalcoholic steatohepatitis (NASH). These two diseases are markedly increasing in the U.S. population, are associated with obesity, and are part of the metabolic syndrome. There is evidence of synergistic effects between steatosis and either excessive oxidative stress, mitochondrial dysfunction, cytokines, altered methionine metabolism, or exposure to industrial pollutants and exacerbation of NAFL/NASH (McClain et al. 2004). In particular, industrial workers exposed to petrochemicals such as benzene, xylene, ethylene, dimethylformamide, or vinyl chloride developed NASH, which resolved with removal from workplace exposure (Cotrim et al. 1999). Recent studies also demonstrate that nutritional intervention, such as antioxidant therapy, can result in significant histologic improvement in NASH (Kugelmas et al. 2003; Louthan et al. 2005). This issue may be especially critical in children and people exposed to environmental toxicants.

Inflammation is an underlying denominator of many diet-related chronic diseases, including cardiovascular disease, diabetes, arthritis, osteoporosis, and cancer. There is evidence that various nutrients and phytochemicals (flavonoids) are associated with a reduced risk of these diseases by affecting underlying molecular mechanisms (Horia and Watkins 2005; Muñoz-Espada and Watkins 2006; Shen et al. 2004). A primary focus of investigation is needed in developing a better understanding of the bioavailability and bioactivity of flavonoids and carotenoids (Manach et al. 2004) to advance the knowledge of diet and foods to alleviate the damaging effects of environmental pollutants and especially POPs. Central to this hypothesis are the proinflammatory properties of omega-6 fatty acids, such as linoleic and arachidonic acids, and the synergistic inflammatory outcomes of fatty acids and POPs (Hennig et al. 2002). The prevalence of environmental toxicants such as heavy metals and organics that contribute to diminished levels of antioxidants will likely aggravate inflammatory states when dietary intakes of omega-3 polyunsaturated fatty acids and polyphenols such as flavonoids are marginal. More research is needed to introduce the concept for studying food components that influence inflammation and how omega-3 polyunsaturated fatty acids and flavonoids could be used therapeutically against inflammation mediated by environmental pollutants.

There is an increased interest in recent years in the health effects of herbal remedies and nutraceuticals in general. For example, there is evidence that polyphenols (and especially green tea catechins) can modulate the absorption of lipids and lipid-soluble compounds (Loest et al. 2002). In fact, evidence indicates that green tea consumption lowers the plasma levels and enhances fecal excretion of lipids and lipid-soluble compounds, including lipophilic POPs (Morita et al. 1997; Yang and Koo 2000). The inhibition of intestinal absorption of lipids by green tea may be associated in part with the inhibition of phospholipid hydrolysis in the intestinal lumen. Further studies are warranted to confirm whether green tea catechins and other flavonoids inhibit the intestinal absorption of lipophilic POPs by inhibiting hydrolysis and micellar solubilization of POPs. Green tea may be recommended as a dietary means of inhibiting the intestinal absorption and enhancing the elimination of lipids and other lipophilic organic compounds, including POPs.

Nutritional intervention has been shown to result in demonstrable improvements in health by lowering the toxicant burden of animals and humans. This was recently illustrated in a case study of a patient who had an extremely high body burden of polychlorinated biphenyls (PCBs) (Redgrave et al. 2005). A fat sample obtained by adipose tissue biopsy revealed 3,200 mg/kg Arochlor 1254. This patient also suffered from diabetes and dyslipemia and required daily injections of insulin. Over approximately 2 years of supplemental consumption of foods containing the fat substitute olestra (fatty acid esters of sucrose; approximately 16 g/day in potato crisps), the PCB body burden of the patient’s adipose tissue dramatically decreased to 56 mg/kg. At the same time, the elimination of the pollutant directly correlated with the disappearance of the patient’s diabetes and normalization of the initial hyperlipidemia. This work, which was also confirmed in animal studies (Jandacek et al. 2005), suggests that *a*) a nonabsorbable oil phase in the intestine reduces absorption of dietary lipophiles, *b*) a nonabsorbable oil phase in the intestine increases the rate of excretion of stored lipophiles that undergo enterohepatic circulation, and *c*) interruption of the enterohepatic circulation can result in clinically meaningful enhancement of excretion of lipophilic compounds.

Another interesting example of effective nutritional intervention is illustrated by the research of Hernandez-Avila et al. (2003), who have extensively studied environmental lead pollution as it affects the maternal and fetal health of populations in Mexico (i.e., women who have had moderately high cumulative lifetime exposure to lead). These researchers discovered that calcium supplementation was associated with a marked decrease in blood lead levels (Hernandez-Avila et al. 2003), as well as breast-milk lead levels (Ettinger et al. 2006) among lactating women over the course of lactation. Furthermore, calcium supplementation during pregnancy decreased maternal blood lead levels and reduced maternal bone resorption. These data demonstrate that nutritional intervention (e.g., calcium supplementation) may constitute an important secondary prevention effort aimed not only at reducing circulating levels of heavy metals such as lead in the mother but also at reducing lead exposure to the developing fetus and nursing infant.

New technologies, such as the “omics” (molecular imaging, nanotechnology, bioinformatics, etc.), present unique opportunities for understanding the molecular mechanism of disease initiation and the underlying effect of nutrition as a mediator of toxicity. Given the rich experimental information on the relationship between reactive oxygen species (ROS) and dietary antioxidants as it relates to human health, there is strong evidence suggesting that bioactive food components can be introduced for prevention and intervention purposes at points of disease initiation and/or progression of pathways leading to an unhealthy or lethal phenotype [for a recent review, see Seifried et al. (2003)]. Unprecedented opportunities exist for the expanded use of nutrition to reduce the risk of disease, and these new enabling technologies would be invaluable in that regard. For example, gene expression studies are providing clues about molecular targets for food components. This may be critical in understanding nutrient/toxicant interactions.

## Conclusions and Recommendations

**Figure 1:**
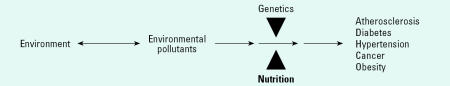
Illustration of the relationship of the toxicology of environmental pollutants to disease and how health effects of exposure can be modulated by both intrinsic and extrinsic factors, namely genetics and nutrition, respectively.

There is a great need to further explore this nutritional paradigm in environmental toxicology and to improve our understanding of the relationship between nutrition, exposure to environmental toxicants, and disease (Hennig et al. 2004). Nutrition may be the most sensible means to develop intervention and prevention strategies for diseases associated with many environmental toxic insults (Figure 1). As discussed above, one of the emerging issues in modern toxicologic sciences is the modification of environmental toxicity by nutrients. Conversely, alterations of the biological or metabolic activity of nutrients by environmental pollutants may be equally important. It is clear that dietary intake and lifestyles can markedly modulate environmental toxicity and associated disease risks. Many disease indicators, such as inflammation and oxidative stress, are known to be influenced by both nutrition and environmental toxicants. This necessitates nutrition as a variable in the study of human disease associated with exposure to environmental pollutants. Humans are increasingly exposed to environmental toxicants, mostly as the result of modern industrial development. Because early nutrition can dictate risk of diseases in the adult population, we strongly recommend the inclusion of nutrition and dietary habits as variables in studies that involve environmental toxicants and disease development. Thus, we recommend that epidemiologic studies on health risks from environmental pollutants also pay close attention to the important role of dietary factors. Prenatal and postnatal exposure to slow-acting environmental factors, including dietary (nutrients) and environmental toxic factors, have the potential to condition adult susceptibility to diseases. The “Precautionary Principle” has been described as the use of comprehensive, coordinated research to protect human health in the face of uncertain risks (Suk and Olden 2004). The NIEHS has taken a leadership role in addressing these uncertainties with an inclusive research approach that includes basic science, as well as translating research findings into public health prevention strategies (Suk and Olden 2004). Nutrition can and should be an integral part of such a comprehensive research approach to protect human health against uncertain risks associated with environmental pollutants.
